# Exploring the adolescents’ career development and career-related teacher support: the NEFELE career guidance model

**DOI:** 10.3389/fpsyg.2024.1437472

**Published:** 2024-11-19

**Authors:** Anna Parola, Federico Diano, Michela Ponticorvo, Luigia Simona Sica

**Affiliations:** Department of Humanities, University of Naples Federico II, Naples, Italy

**Keywords:** career construction, career guidance, career adaptability, career exploration, adolescents, teachers, game-based approach, randomized control trial

## Abstract

**Introduction:**

In today’s dynamic landscape, navigating career paths in the midst of economic, societal, and technological changes has become increasingly challenging. The developmental psychology studies (see the career construction theory framework) and European educational standards consistently support the crucial role of teachers in supporting students’ career choices. However, teachers do not seem to be trained for this role, and the NEFELE project aims to fill this gap. The training model proposed in this paper aims to provide teachers with the knowledge and tools to support their students’ career choices in educational systems.

**Methods:**

Using a randomized control trial, a study was conducted in the Italian context involving 8 teachers and their classrooms. Pre- and post-test measurements were carried out to study the effectiveness of the intervention.

**Results:**

Students in the experimental group who participated in career guidance activities taught by teachers trained through the NEFELE training model show an increase in career-related teacher support, career adaptability, career explorations, and career competences.

**Discussion:**

Implications for practice are discussed.

## 1 Introduction

Nowadays, careers unfold in an unpredictable environment characterized by current economic, social, and technological challenges. The nature of employment has undergone dramatic changes, and in most European contexts, young people are the most vulnerable category to unemployment (International Labor Organization, 2020). The NEET rate, i.e., people not engaged in education, employment or training, in the European Union has fluctuated over time and differs between Member States (Eurostat, 2020). In recent years, it has been around 11–12% for people aged 15–29, but this can vary from country to country. Countries such as Italy, Spain, Greece, and Portugal have historically faced higher NEET rates than the EU average. These countries suffer from a stagnant economy, which has a significant impact on youth employment opportunities, which in turn affects career choices.

In addition, the impact of megatrends such as the digitization of products, services and processes, the digital transformation of the labor market through, for example, automation, increasing occupational mobility, and demographic shifts have led to changes in economic, social, and environmental spheres. These changes have a direct or indirect impact on workers, enterprises and training systems (Organization for Economic Co-operation and Development, 2018). This evidence has brought an ever-increasing need to acquire knowledge and skills to keep up with the technological changes needed to transition to the changing world of work. Other changes also deserve to be addressed: wars, pandemics, and environmental changes that inevitably raise fears about the future yet to be shaped. Such complexities inevitably affect adolescents’ and young adults’ visions of the future, leading to worry and distress (Regnoli et al., 2024). Environmental uncertainty leads to fragmented career paths (Baruch and Bozionelos, 2011) and involves the management of increasingly uncertain career paths with difficulties in the career decision-making process (Guichard and Di Fabio, 2015). Career choices are among the most important decisions individuals make in their lives (Bimrose and Mulvey, 2015). According to Kulcsár et al. (2020, p. 2), and colleagues career decision-making “may involve choosing an occupation and the educational training involved, then a job and then whether to stay at a job or switch to another one, what formal and informal advanced training to take, and so on. When facing such decision, many individuals experience difficulties that often prevent making them or lead to choosing a non-optimal alternative.”

In light of these changes, it seems necessary to help adolescents and young adults navigate “uncertain and unpredictable waters.” This context of difficult entry into the job market leads adolescents and young adults to postpone their process of vocational identity formation, postponing identity commitments and future planning (Fusco et al., 2022). The education system, which is responsible for the human and educational growth of individuals, is called upon to provide students with the tools to facilitate career choices.

This paper presents a career guidance model that can be implemented in the education system, including teacher training and career guidance tools. The proposed career guidance model is anchored in the interplay between career construction theory (Savickas, 2011) and positive education (Waters and Loton, 2019). The model is the result of the Erasmus+ KA2 NEFELE project, which involved several European countries: Italy, Spain, Greece, Switzerland, and the Netherlands. This paper focus specifically on the piloting of the whole model in the Italian context.

### 1.1 Career development: the life-span perspective

Career development should be conceived as a lifelong process spanning infancy through childhood, adolescence, adulthood, and old age (Hartung et al., 2005; Magnuson and Starr, 2000). As Hartung et al. (2005) and colleagues suggested, career development begins much earlier in the lifespan, with children’s early perceptions of work shaping their choices as they transition into adolescence and young adulthood.

The formulation of the career concept begins around ages 8–9, when children develop an understanding of work through practical observation and recognize its role in supporting the family (Schultheiss et al., 2005). They also begin to understand the positive and negative aspects of work, such as fatigue (Schultheiss, 2008). Consistent progress in career exploration, aspirations, interests, and adaptability between the ages 9–10 facilitates the career decision-making process and fosters environmental connectedness (Ferrari et al., 2015; Hartung et al., 2005; Magnuson and Starr, 2000; Watson and McMahon, 2008). Career activities during this stage serve a preventive purpose (Meijers and Lengelle, 2012). This period should be viewed as an active engagement in career exploration engagement to cultivate career adaptability, instill confidence in future career choices, foster a sense of control over one’s life, and shape ideas about career choices.

During adolescence, which typically begins around ages 11–12, future orientation skills develop along with increased independence and personal identity construction. Adolescents begin to delineate g more detailed future goals, particularly in the areas of educational and career, and relate them to real-world contexts (Arnett, 2000). In the early stages of the exploration process, it is crucial to stimulate reflection on what children observe and to provide diverse examples of activities to support their education and stimulate their interests (Ferrari et al., 2015). In addition, emotional support and role modeling from by parents and teachers play critical roles in this life stage, facilitating adolescents’ career exploration and information gathering (Taveira and Moreno, 2003).

Since individuals in this age group typically attend middle school, these years are crucial for career development, future planning, and emotional well-being. Middle schools need to serve as important periods for fostering career development, instilling hope and optimism about the future, and mitigating feelings of fear, distress, and anxiety that may overshadow adolescents’ views on their futures. According to Maree (2021, p.444), promoting “career development in people’s early years should help learners design themselves and choose subjects, fields of study, and careers that will not only ‘fit’ their profiles but help them enact key life themes in their career-lives and experience meaning, hope, and purpose in their future careers.”

Career Construction Theory (CCT; Savickas, 2012) provides a theoretical framework for understanding the interpretive and interpersonal processes by which individuals construct their own selves, give direction to their vocational behavior, and make sense of their careers. During adolescence, key developmental tasks include individuals viewing work as a salient role, crystallizing preferences for vocational fields, specifying career preferences, and transition to an appropriate career (Savickas, 2012). Career adaptability refers to psychosocial resources for coping with vocational development tasks (Savickas, 1997). Specifically, career adaptability is considered a psychosocial resource because it regulates the relationship between the individual and their environment. The CCT defines four global dimensions of career adaptability and organizes them into a structural model with three levels. At the highest level are placed the four dimensions of career adaptability, i.e., concern, control, curiosity, and confidence, which allow individuals to cope with vocational tasks. At the intermediate level are the ABCs of career construction: attitude, beliefs and competencies. Disposition attitudes and beliefs influence the development and use of competencies. Consequently, cognitive competencies influence the adaptive behaviors that actively facilitate career development and career construction. On the third level are the coping behaviors that make up the adaptive functions, i.e., orientation, exploration, establishment, maintenance, and disengagement.

### 1.2 The interplay between career construction theory and positive psychology

Positive psychology is a holistic framework that helps address the well-being and new challenges faced by adolescents. Specifically, positive psychology explains the conditions and processes that contribute to the flourishing or optimal functioning of individuals by building on their strengths and virtues (Gable and Haidt, 2005). Positive psychology aims to promote positive emotions, positive relationships, character strengths, and, in general, the development of skills to achieve happiness and well-being. Six elements are framed in the character of strengths framework of the Values in Action (VIA) Classification of Strengths (Peterson and Seligman, 2004). The model emphasized the need to nurture, use, and grow character strengths. Character strengths are defined as traits or attributes that individuals hold morally valuable. These positive traits influence how individuals think, feel, and behave in various domains and contribute to personal growth, well-being, and success. The VIA model also provides a flexible approach to planning, implementing, and evaluating positive psychology in the school setting, including explicit and implicit instruction.

The literature supports the interplay between CCT and positive psychology (Robertson, 2018; Parola and Marcionetti, 2022; Magnano et al., 2021). Some studies have shown how focusing on strengths can help with future orientation (Parola et al., 2024a) and promote well-being and job satisfaction (Littman-Ovadia and Davidovitch, 2010; Dik et al., 2015). Owens et al. (2016) highlighted that it can be helpful in university counseling contexts to promote career decision-making self-efficacy.

Cultivating character strengths in educational contexts can help students develop a deeper understanding of themselves, make career choices that fit with them and ultimately lead fulfilling and purposeful lives.

### 1.3 Drawing the future in educational contexts

In recent years, there has been an emphasis on supporting students’ career development within educational systems (Parola et al., 2023; Sgaramella and Ferrari, 2024). Given that children and adolescents spend a significant portion of their time in school, teachers have considerable potential as key sources of support (Metheny et al., 2008). In the face of today’s challenges, teachers are critical in “scaffolding” students’ career transitions. The education system must successfully equip students to manage these transitions by providing new and useful skills, competencies, and qualifications (Marcionetti and Rossier, 2016). Individuals are increasingly empowered to shape their careers, determine their life paths, and define their societal roles (Cohen-Scali et al., 2018). As recommended by several studies, fostering a sense of curiosity and exploration along with positive attitude toward career development have become central to adolescents’ (Fusco et al., 2021; Koivisto et al., 2011; Parola et al., 2023). In addition, life skills such as decision-making, problem solving, and critical thinking are essential for adolescents to navigate career choices in an increasingly complex world (Commodari et al., 2022; Salonen et al., 2017). As shown in a recent study by Pedditzi et al. (2023), students’ self-efficacy in life skills is most related to the age at which the school transition occurred, suggesting the importance of fostering the development of this dimension to support students during school transitions.

Nevertheless, the traditional school curriculum is not always aligned with future job demands (Economist Intelligence Unit, 2018) and does not appear to provide useful resources for transitioning adolescents. According to a recent study conducted by the OECD and WorldSkills (2019), young people report a lack of support from the education system, with 44% expressing concerns about the relevance of their skills and knowledge to the future job market.

As a frontline figure in the school, teachers play a pivotal role in nurturing students’ career skills and fostering positive outlooks. Career-related teacher support refers to the teachers’ behavior toward students regarding information and support for career development (Wong et al., 2021; Parola et al., 2023). Metheny et al. (2008) identified four teacher career attitudes: invested effort, positive regard, positive expectations, and accessibility. Invested effort refers to the teacher’s willingness to take action to promote student success. Positive regard indicates that teachers are emotionally connected to their students and are responsive to students’ needs. Positive expectations involve teachers conveying optimism about students’ future academic and career achievement. Finally, accessibility means that students view teachers as approachable and readily available for help or information.

Studies on the relationship between career-related teacher support and adolescent career outcomes are limited. The literature has shown an effect on career readiness (Perry et al., 2010), career adaptability (Hlad’o et al., 2020), and career decision self-efficacy (Gushue and Whitson, 2006).

Recently, Sgaramella and Ferrari (2024) systematized studies conducted to promote career adaptability resources in adolescents. Guided by CCT, the studies proposed interventions with reflective exercises (e.g., Gülşen et al., 2021; Santisi et al., 2021) through videos and guidance booklets. Content included self-knowledge and context knowledge (barriers and supports). Sgaramella and Ferrari (2024) emphasized the importance of working in the educational context on knowledge and self-knowledge to promote career readiness.

Despite the recognized importance of teachers in career development, they often spend more time to addressing learning difficulties rather than guiding students in making informed career choices. In addition, many teachers are not adequately trained for this role.

## 2 NEFELE career guidance model

The NEFELE Career Guidance Model aims to address two levels:

1. Filling the gap in teachers’ knowledge about students’ career construction by providing theoretical knowledge and practical tools for career guidance functions in the classroom.
2. Designing a career guidance model for adolescents that is explicitly tailored for educational settings and takes into account the student-teacher relationship.

To achieve the first goal, NEFELE offers a teacher training course, “Promoting Students Career Development at School,” through Massive Open Online Courses (MOOC). The NEFELE is based on Moodle 4.0, and it was developed following a collaborative team-based methodology for MOOC development based on the ADDIE model (Analysis, Design, Development, Implementation and Evaluation) (Molenda, 2003). The lessons aim to provide users (pre-service and in-service teachers) with the knowledge and career guidance tools necessary to support students’ career development while promoting their well-being. The MOOC consists of five modules with recorded online lectures, slides, and learning materials. Teachers will learn about career development from childhood through adolescence in the life span perspective, career construction theory and career guidance models (i.e., the life design), and positive psychology theories. In addition, teachers will gain insight into the practical competences that students need to make career choices. They will be trained in digital, green, entrepreneurial and life skills according to European frameworks (DigComp, GreenComp, EntreComp, LifeComp). Completion of the module after passing the self-assessment leads to the acquisition of badges.

For the second goal, NEFELE designed a career guidance model specific to the educational context. Teachers trained through MOOCs will be able to guide students through career exploration, focusing on their strengths and acquiring the skills needed to face current challenges. The classroom career guidance model uses a game designed to stimulate career exploration. The NEFELE digital game represents a set of tools to support career development through co-creation and gamification. Specifically, the NEFELE game aims to support career development and promote career exploration through gamification and storytelling, enhanced by Tangible User Interfaces (TUIs) and NFC technology tools.

From a theoretical point of view, the NEFELE game follows the Life-Span Perspective of career exploration developed initially by Super et al. (1996) and Turner and Lapan (2013) and extended by Lent and Brown (2013). In this perspective, career exploration can be understood as a continuous process that take place throughout the lifespan and as an adaptive mechanism that helps to cope with environmental changes (Blustein, 1997; Zikic and Klehe, 2006). Adolescents and young adults can explore their careers by reflecting on internal and external factors (Jiang et al., 2019). In career construction theory, career exploration is seen as a competence (intermediate variable) related to curiosity (Savickas, 2012). In this sense, increasing curiosity through play could enhance students’ career exploration. With this in mind, the NEFELE game was designed to allow adolescents to reflect and “play” with, on the one hand, with the competences needed for the transition to the world of work and, on the other hand, with personal resources, such as career adaptability and character strength, in order to enhance career exploration.

From a technological point of view, NEFELE uses TUIs technology. TUIs are user interfaces that allow individuals to interact with digital information through physical environments (Roebuck, 2011). Several studies have suggested the usefulness of using TUIs in educational settings (for a review, see Rodi and Grani, 2022) to encourage exploratory and expressive activities (Marshall, 2007). In the NEFELE game, a hybrid approach combines digital and tangible technologies to create an overall experience based on the strengths of both approaches. Traditional games and experiences can be enhanced to achieve greater results, thanks to the higher engagement and motivation supported by specific technological and tangible tools.

The game consists of (a) physical materials: the game board and cards, which are enhanced with NFC sensor tags and thus became smart objects, (b) the editor for creating the career stories; (c) the game app. The game board consists of five “rooms”: Digital Competences, Entrepreneurial Competences, Green Competences, Life Competences, and the “If I Were” room. For the competency rooms, the user has to imagine a character’s competences and their involvement in the chosen job. For the “If I Were” room, users will try to describe themselves in the role of the selected job, linking their strengths and values with the ultimate goal of setting personal and career goals. The ultimate goal is to help users imagine different versions of the same job, based on different personal resources. The cards represent potential jobs (30 representatives of ESCO categories and 10 customizable cards), competences, strengths, and values.

Students, individually or in groups in the classroom, can imagine individual competences and characteristics that can play a decisive role in a job. The editor will allow them to register and associate their description with the job and then be able to replay it in the form of a riddle within the APP. The APP will allow them to play with previously created and default scenarios. Using the clues, students will form teams to play and challenge each other to guess a character’s job. In addition, the APP has three sections that provide educational materials for users: Support, Self-Care, and Learning Materials. The Support section will identify key figures that adolescents can turn to for help in the career choice process. In the Self-care section, students will find information on psychological strategies they can practice to increase their well-being. Finally, the learning materials link to competence fact sheets. These materials are also useful for teachers who use the game in a laboratory format in the classroom.

This innovative tool allows students to experiment with themselves in different roles, such as jobs, opening up to a positive vision of the future, curiosity as a resource for adaptability and improving planning and future orientation skills. In this sense, adolescents can construct their content in an authoring system, and this game is an elective tool to engage them in career exploration. The game can be used in schools and played in classroom teams. The teacher acts as a guide and support.

## 3 NEFELE trial: study aims and contextual location

**Figure 1:**
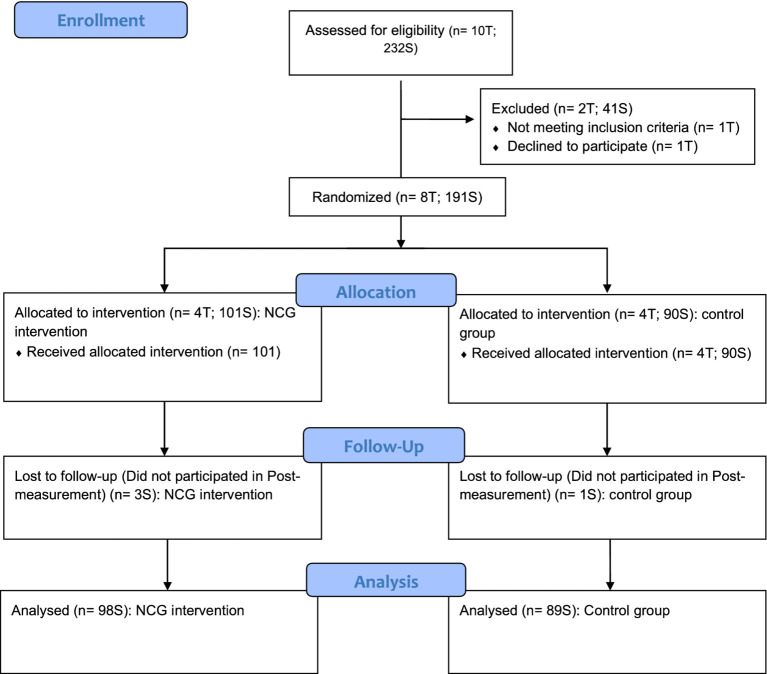
Consort flow diagram for randomized control trial. NCG = NEFELE career guidance intervention. For each assignment: the teacher was assigned to the condition. Each teacher involved the class in the experimental (CCG) and control group (the number of students depends on the size of the teacher’s class in the condition). T, teachers; S, students.

Using a randomized control trial (RCT) design, this study aims to examine the effects of the NEFELE career guidance model on students’ career-related teacher support, career adaptability, career exploration and career competences. A total of 8 middle school teachers with their respective students (4 classroom per teacher) were randomly assigned to two conditions: NEFELE career guidance intervention (NCG), and general career guidance intervention (GCG). These two interventions were compared to a control group with absence of conditions as usual. For details of the conditions, see the Participants and Study Protocols section and the Figure 1.

The following research questions and hypotheses were addressed: Can adolescents’ career-related teacher support, career adaptability, career exploration, and career competences be promoted through a NEFELE training model that includes a teacher trained in this function? It is expected that the intervention model (with trained teachers) will promote career-related teacher support, career adaptability, career exploration and career competences.

The study was conducted in the Italian context. In Italy, middle schools last 3 years, at the end of which adolescents are still in compulsory education and have to choose which high school to attend. The choice of high school could be crucial for the subsequent choices that adolescents will make. Previous research conducted in the Italian context (Parola and Marcionetti, 2021) has shown that career indecision among middle school students is high and that the role played by external factors (e.g., parents) seems to be crucial. Schools, unlike other countries, do not provide career guidance provided by career practitioners (see, for example, best practices in the Swiss context in Parola and Marcionetti, 2020). The most recent Italian jurisdiction (December 2022) considers the role of the teacher crucial by introducting 30 h of “guidance didactics” (in Italian, “Didattica Orientativa”) within the classroom of middle and high schools. Unfortunately, teachers are left alone and have no guidelines to follow.

## 4 Methods

### 4.1 Participants and group protocols

The flow diagram of participants is displayed in Figure 1. The interventions were carried out in the middle schools of Southern Italy. School principals and teachers were invited to dissemination events of the NEFELE career guidance model targeted at middle school teachers during the second half of 2023. After the events, an email was sent to the schools asking about the possibility of conducting the project pilot with their in-service teachers, and 12 teachers responded positively. A researcher conducted a briefing with the teachers and check the eligibility criteria. A total of 8 teachers and 4 classrooms per teacher participated in the study and were assigned to two groups: (1) NEFELE Career Guidance (NCG); (2) Control Group (CG), which received no additional support for this career guidance function as usual.

#### 4.1.1 The NEFELE career guidance (NCG) group

The NCG intervention group includes 4 teachers who participated in the entire NEFELE training model including: full training on the 5 modules via MOOC (which includes training on the digital game). After the training, the teachers were invited to use the digital game in their 4 classes (*n* = 98 students, 42 males and 56 females). The students were involved in the pre- and post-assessment.

#### 4.1.2 The control group (CG)

The control group received no training model for teachers and no interventions for students. 4 teachers and their 4 classrooms (*n* = 89 students, 41 males and 48 females) were involved. Teachers were asked to conduct routine career guidance activities as usual. Students were involved in pre- and post-assessment.

### 4.2 Measures

#### 4.2.1 Career-related teacher support

The Career-related Teacher Support (CRTS; Wong et al., 2022; Italian validation by Parola et al. (2024a)) was administered to capture students’ perceived support from teachers. This instrument allows for the specific investigation of perceived support in relation to career choices. This instrument is composed of 20 items and five dimensions: Positive Expectations (5 items), Motivational Support (5 items), Informational Support (4 items), Accessibility (3 items), and Positive Regard (3 items). Students were asked to respond on a scale 5-point Likert-type scale ranging from 1 (= never) to 5 (= always). Typical items include “My teachers think I should continue my education after secondary school” and “My teachers are easy to talk to out of lesson time.”

#### 4.2.2 Career adaptability

The Career Adapt-Abilities Scale (CAAS; Savickas, 2012; Italian validation by Soresi et al. (2012)) was used to assess career adaptabilities. This instrument is composed of 24 items and four dimensions: Concern (6 items, e.g., “Becoming aware of the educational and career choices that I must make”), Control (6 items, e.g., “Taking responsibility for my actions”), Curiosity (6 items, e.g., “Exploring my surroundings”), and Confidence (6 items, e.g., “Working up to my ability”). Students were asked to respond on a scale 5-point Likert-type scale ranging from 1 (= not strong) to 5 (= strongest).

#### 4.2.3 Career exploration

To measure career exploration, following the measure proposed by Marcionetti and Rossier (2016), six career activities were proposed to students (e.g., “Think for yourself about which educational/professional path to pursue”). Individual score was obtained by summing the activities reported by students at the time of the questionnaire.

#### 4.2.4 Career competences for future

An *ad hoc* instrument consisting of 5 items was created to explore the propensity to consider that European skills are relevant to future educational and career choices. Participants were asked to indicate the influence of each competency domain (e.g., “Digital Competences”) on their educational and career choices on a five-point Likert scale ranging from 1 (= almost not at all) to 5 (= very much). As the instrument was created *ad hoc*, the one-dimensional factorial structure of the instrument was checked prior to its use in the data analysis. Confirmatory factor analysis (CFA) with robust maximum likelihood (MLR) estimator shows a good fit of the model to the data [*χ*^2^ (5) = 6.21, *p* = 0.250, CFI 0.990, TLI = 0.980, RMSEA 0.042, SRMR 0.032].

## 5 Data analysis

First, the differences in the pre-measurement between the student participants for each group were assessed through *t* test. Descriptive analysis, i.e., means and standard deviations, and the check of skewness and kurtosis to the assumption of normality were performed. There were no missing data for any of the participants on any of the measures at any of the measurement points (pre-post). Reliability was assessed by internal consistency analysis, using Cronbach’s alpha (*α*) at each of the measurement points (pre-post).

Differences in the changes in different aspects of career-related teacher support, career adaptability, career explorations and career competences were examined through repeated measures analysis of variance (RM-ANOVA). The unbiased sample estimate standardized mean difference effect sizes (Hedges’ *g*; Hedges, 1981) was used to evaluate the magnitude of change from pre- to post-measurement. The following ranges were used to interpret the magnitude of the differences: from 0.20 to 0.49 = small; from 0.50 to 0.79 = medium; from 0.80 = large (Cohen, 1988).

## 6 Results

**Figure 2:**
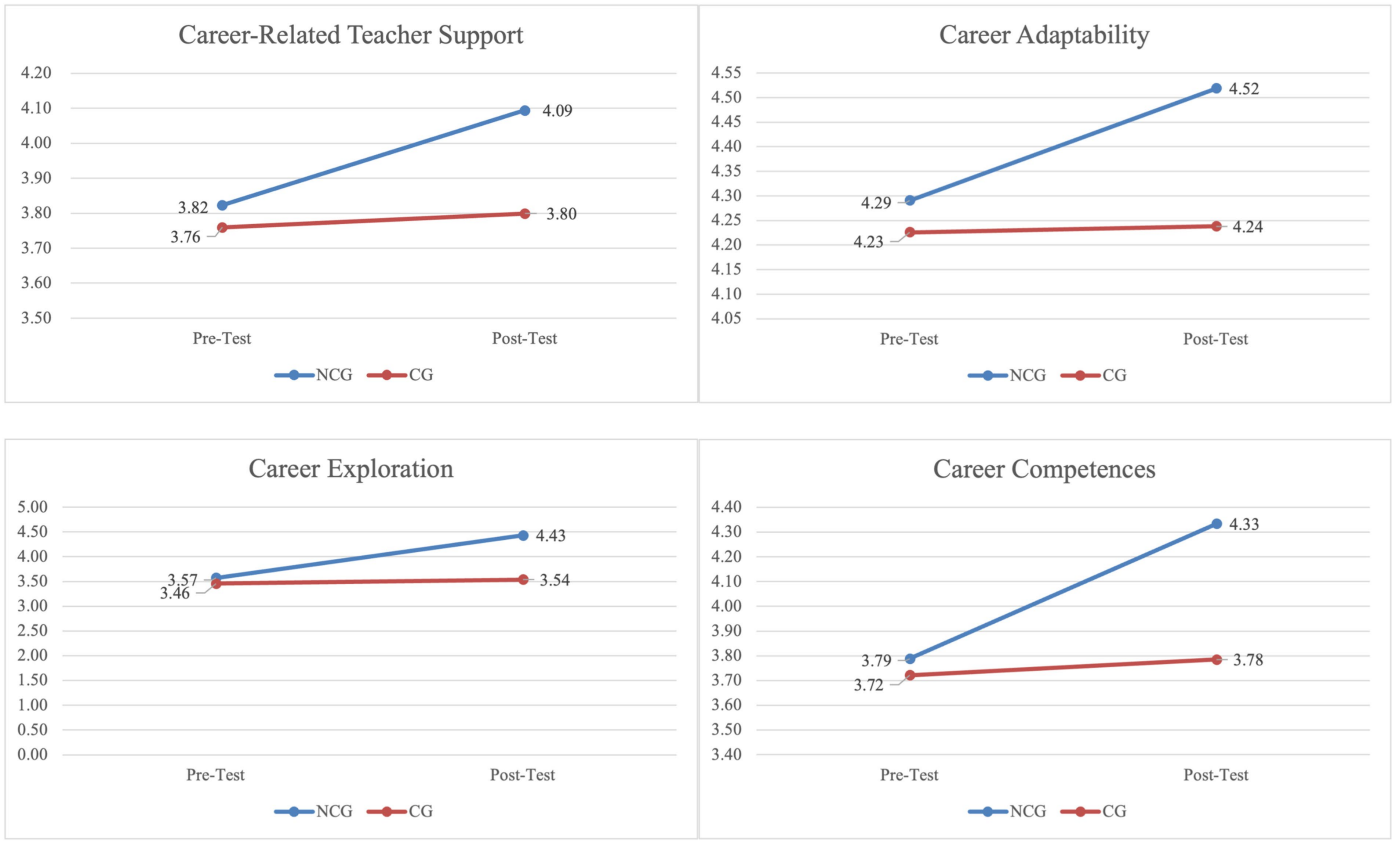
Means of the career dimensions pre- and post-intervention for each condition (experimental and control group).

Means and standard deviations between the two time intervals (T1, T2) are displayed in Table 1. The Hedges’ *g* values are shown in Table 1. A representative graph of the distribution of the means for the NCG and CG groups from T1 to T2 are displayed in Figure 2.

**Table 1:** Mean, standard deviation, reliability coefficients and effect size (|g|) for each time comparison.

	Descriptive		Reliability (Cronbach’s α)		Time comparison (Hedge’s g)	
	M (SD)	M (SD)				
	T1	T2	T1	T2	T1	T2
Career-related teacher support						
NCG	3.823 (0.622)	4.094 (0.693)	0.948	0.942	-	0.326
CG	3.759 (0.627)	3.799 (0.580)	0.939	0.938	-	0.066
Career adaptability						
NCG	4.290 (0.528)	4.518 (0.546)	0.958	0.934	-	0.422
CG	4.226 (0.506)	4.238 (0.529)	0.947	0.942	-	0.096
Career exploration						
NGG	3.571 (0.788)	4.431 (0.777)	0.813	0.824	-	1.095
CG	3.457 (1.002)	3.537 (1.049)	0.817	0.820	-	0.077
Career competences						
NCG	3.788 (0.891)	4.333 (0.618)	0.881	0.905	-	0.708
CG	3.721 (0.887)	3.784 (0.903)	0.882	0.892	-	0.070

NCG, NEFELE career guidance; CG, control group; M, mean; SD, Standard deviation; T1, Time 1 (pre); T2, Time 2 (post).

The pre-measurement *t*-test showed no differences in career-related teacher support, career adaptability, career exploration and career competences at the pre-measurement (all *ps* < 0.001). The analysis indicated that the groups were homogeneous before the intervention (at baseline).

A repeated-measures ANOVA was conducted to examine the interaction effect between group (NCG vs. CG) and time (pre-test vs. post-test) on career-related teacher support, career adaptability, career exploration and career competences. The results of the repeated-measures ANOVA reveal a significant time interaction [*F* (1,185) = 38.311, *p <* 0.001, *η*^2^ = 0.172] and group x time interaction [*F* (1,185) = 21.197, *p* < 0.001, *η*^2^ = 0.10.] for career-related teacher support. The effect highlighted a significant increase of career-related teacher support from T1 to T2 in the NGC group. The Hedges’ *g* value comparing T1 and T2 was 0.326.

A significant time interaction [*F* (1,185) = 21.372, *p* < 0.001, *η*^2^ = 0.104] and group x time interaction [*F* (1,185) = 17.259, *p* < 0.001, *η*^2^ = 0.985] for career adaptability emerged, showing a significant increase of career adaptability from T1 to T2 in NGC group. The Hedges’ *g* value comparing T1 and T2 was 0.422. Moreover, a significant time interaction [*F* (1,185) = 83.766, *p* < 0.001, *η*^2^ = 0.312] and group x time interaction [*F* (1,185) = 57.580, *p* < 0.001, *η*^2^ = 0.237] emerged for career exploration. The effect highlighted a significant increase of career-related teacher support from T1 to T2 in the NGC group. The Hedges’ *g* value comparing T1 and T2 was 1.095. Finally, the results of repeated-measures ANOVA reveal a significant time interaction [*F* (1,185) = 47.343, *p* < 0.001, *η*^2^ = 0.204] and group x time interaction [*F* (1,185) = 29.726, *p* < 0.001, *η*^2^ = 0.138] also for career competences showing higher career competences between time intervals in the NCG group. The Hedges’ *g* value comparing T1 and T4 was 0.708.

## 7 Discussion

This aim of this work was to present the NEFELE training model and evaluate its effectiveness on a group of middle school students in Italian country. The NEFELE training model provides middle school teachers with theoretically and practically training in career guidance. Equipped with the knowledge and practical tools, teachers will be able to perform the school duties of “career practitioners” as required by Italian national standards and be effective in their role. Therefore, this paper aims to fill two gaps: on the one hand, the one recognized at the EU level but also in national school policies of the lack of Continuing Professional Teacher Development (CPTD) qualifications and/or courses that support teachers in acquiring the necessary skills for this role; and on the other hand, this work makes it possible to expand the literature on the effectiveness of guidance interventions offered by teachers in middle school classrooms.

Although the training model has been designed at the EU level, the trial has so far only been carried out in the Italian context. The teachers involved in the trial have in turn involved their students. The teachers were randomly assigned to an experimental group and a control group. As mentioned above, the teachers assigned to the experimental group received 5 weeks of training through a NEFELE MOOC and then used the NEFELE digital game. Teachers assigned to the control group did not receive the training and were required to carry out normal classroom activities. A pre-post administration made it possible to evaluate the effectiveness of the training model on the students. Specifically, the NEFELE trial aimed to investigate whether a teacher trained in this area could increase career-related teacher support, career adaptability, career exploration and career competences among middle school students.

A pre-post comparison between the experimental and control groups was conducted using RM-ANOVA. As hypothesized, the results clearly show the effectiveness of the NEFELE career guidance on the career outcomes targeted in this study. The significant differences between the students’ pre-and post-test scores show an increase in all the dimensions addressed, i.e., career-related teacher support, career adaptability, career exploration, and career competences.

Career-related teacher support refers to motivational support, informational support, positive teachers expectations have toward them, perceived availability when needed, and emotional support (Parola et al., 2024a; Wong et al., 2021). Consistent with the literature on career-related teacher support, adolescents perceive teachers as a reference figures for their career construction (Dick and Rallis, 1991; Yang and Wong, 2020), and role models who influence career decisions (Betz, 1994), and sources of career information (Hirschi, 2012; Wong et al., 2019), which influences the formation of positive behaviors and career expectations (Farmer et al., 1995). The results of this study suggest that a trained teacher is even more likely to be perceived as a protective factor for the student, likely because the teacher, in turn, has been trained to have the tools to help students develop their future career plans.

The increase in career adaptability, exploration, and competences in the experimental group is in line with the dimensions that the NEFELE training and the game aim to improve. Indeed, the game is a tool in the hands of the teacher, who has the function of ‘game master’. This aspect is of fundamental importance because career-related teacher support is associated with several positive career outcomes, such as career decision-making self-efficacy (Parola et al., 2024a), career preparation (Perry et al., 2010), and motivation to pursue their career goals (Phillips et al., 2002). The pilot activities of the RCT involved the use of the NEFELE Box in a classroom setting, where students engaged in role-playing and scenario-based games to explore different career paths. This activity used job cards enhanced with NFC sensor tags representing various professions, such as medical doctors, engineers, and artists. Students interacted with these cards through the NEFELE game app, which provided detailed job descriptions and skill requirements, prompting them to think critically about the skills and educational pathways needed for each career. Imagining themselves in a profession or guessing someone else’s chosen career is intended to facilitate reflection on the abilities needed for career transitions (career adaptability) and to consider competences such as digital skills as crucial for future career choices in the perspective of sustainable and inclusive careers.

Furthermore, playing with professions allows for an increase in career exploration processes. The results of dimensional change (Hedge’s g) show a significant increase between the pre-test and the post-test for career exploration. This result demonstrates that it is possible to stimulate career exploration through specific career guidance activities, such as the digital game, that increase the possible alternatives related to the possible selves (Markus and Nurius, 1987) that adolescents aspire to become. Game-based career guidance emerges as an important trigger for career construction. The gamification element would allow young people to imagine themselves in the future by experimenting with different roles (Parola et al., 2024b). Resource enhancement through the NEFELE game and mediated by the trained teacher therefore appears to be a valid way to support young people in their career development. This finding supports the literature that considers the interplay between career development and positive psychology as fertile ground on which to base guidance interventions aimed at promoting ‘adaptive’ transitions (Parola et al., 2023) between education systems and the world of work.

The NEFELE model proposed to teachers is aimed precisely at communicating to students their willingness to help them make career choices, whether it be motivational, informational, or emotional, but also to discuss possible alternatives for the future and to imagine careers tailored to them. These careers can take into account interests, competences, personal abilities, values and character strengths. In contrast to a “matching people to jobs” approach, the NEFELE model aims to open students’ minds to all possible career paths.

### 7.1 Limitations, strengths and future research directions

The study is not free of limitations. First, the study was conducted only in the Italian context. Future research efforts will need to include other European countries to determine the effectiveness of the program in different contexts. As mentioned above, the NEFELE training model was developed as part of the Erasmus+ NEFELE project funded by the European Community, which also involved the countries of Spain, Greece, Switzerland, and the Netherlands. Future studies will be designed to compare the results of this Italian pilot project with career development projects or other countries, in order to highlight both the transferability and the specificity of the model.

Second, assessing the effects of the digital game was not feasible within the current design. Indeed, the study does not isolate the effects of the NEFELE on career-related outcomes. To address this issue, future studies should use a randomized design with three different conditions: an experimental group following NEFELE training model via MOOCs and using the game (first condition), a second experimental group receiving MOOC training but without access to the digital game (second condition), and a control group as usual (third condition). The lack of such a design in this study was due to the fact that the NEFELE MOOC included game training in its last two modules. Pordelan et al. (2021) demonstrated that digital storytelling in career guidance led to better outcomes in students’ career decision-making self-efficacy compared to groups receiving face-to-face life design programs. Therefore, future studies should also include a training-only group that completes only the first two MOOC modules to isolate the impact of the NEFELE digital game on career outcomes.

Third, the study focused only on students in the middle of the third-grade, who are ready to transition to high school. Future research could examine the effectiveness of the model in the early years of high school. Fourth, follow-up assessments were not conducted. Future studies should evaluate long-term outcome measurements to assess the interventions’ sustained effects. A follow-up study at 6- and 12-month intervals would be helpful to observe sustained outcomes in career adaptability and career explorations.

Fifth, the trial relied on self-report measures for data collection. Future research should integrate additional data collection methods, such as observation and/or interviews, for example in the classroom. Finally, due to the small number of participants, it was not possible to examine the effect of the teacher (e.g., skills acquired, self-efficacy) on the classroom. Future studies will consider the possibility of conducting multilevel analyses involving more classes to also assess the teacher-class relationship.

Despite these limitations, the NEFELE model has several strengths. First, compared to previous interventions in educational systems, this intervention assumes the active involvement of the teacher after specific training. Second, it introduces the gamification aspect into the life design model. A recent review showed that there has been an increase in digital games in classrooms in recent years, but games for guidance often lack a solid theory (Parola et al., 2024b). The lack of a clear theory and the dimensions of the career that the game is intended to improve risks not allowing a clear evaluation of its effectiveness. Finally, the effectiveness of this training model on students has been demonstrated by RCT. This is the most rigorous and robust research method for determining whether a cause-effect relationship exists between an intervention and outcomes (Bhide et al., 2018).

### 7.2 Implications and policy recommendations

These results open up interesting implications at different levels. In term of practical implications, the study shows how training models aimed at improving teachers’ knowledge have cascading effects on students. From this point of view, planning interventions to support teachers in this function by providing them with a solid theoretical framework and tools to use in the classroom is the starting point for implementing effective interventions in educational settings.

Thus, institutional policy recommendations can be made. Middle schools should integrate career guidance into their curriculum and ensure that every student has access to comprehensive career guidance. This includes training teachers to incorporate career education elements into their lessons, regardless of the subject matter. This initiative implies that educators must be trained to guide students in making informed career decisions and to integrate these competencies into everyday teaching practices in all educational settings.

Guidelines for teachers’ practice of career guidance are needed to achieve interventions based on students’ needs, starting from a sound theoretical framework. According to a recent article by Van Hai et al. (2022), factors that influence the effectiveness of career guidance and counseling for middle school students include the teachers’ qualification, professionalism, and sense of responsibility of teachers. Providing teachers with the theoretical foundations and tools to implement career guidance activities is a prerequisite for positive career outcomes for students. Indeed, intervention programs should not be ‘improvised’, but should respond to solid theoretical frameworks that also allow for the design of evidence-based interventions.

Finally, the digital game can be used in different settings. The first is the educational setting. The game can be used in schools and played in classroom teams. The teacher who plays a key role in students’ career exploration could serve as a guide and support. In addition, the game could be used in career counseling practices. The career counselor could use it to stimulate career exploration in adolescents through career digital storytelling (Parola et al., 2022). Finally, the game could be used at home, with parents, siblings, and friends to enhance career explorations.

## 8 Conclusion

The NEFELE training model combines the latest in educational technologies and methodologies, such as MOOCs and TUIs, to provide innovative, interactive learning experiences that help adolescents understand and develop skills to meet future labor market demands, thus helping them to start building their careers.

The key recommendations of the NEFELE project strategically advocate for the integration of this career guidance model into mainstream curricula and teacher training programs. This strategic integration will significantly improve the educational framework and effectively reduce the incidence of NEET status among young people by equipping them with the skills and perspectives necessary for future career success.

The paper demonstrated the need to train teachers in the function of career counselors to better serve students. Trained teachers using activities such as the NEFELE game with a solid theoretical framework can achieve improvements in important career outcomes such as career exploration, career adaptability, and competencies. These are indeed necessary for adaptive transitions between educational systems and also for school-to-work transitions. Stimulating career exploration should be the responsibility of education systems, and the NEFELE model aims to train teachers to stimulate it in the classroom.

Finally, higher education institutions should include training in this function for pre-service teachers in their curricula. In parallel, education systems should include structured career guidance programs and provide in-service teacher training in this area.

## Data Availability

The raw data supporting the conclusions of this article will be made available by the authors, without undue reservation.
